# Supplementary material for: Molecular crowding effects on protein stability in a bacterial proteome
Source: Sci Rep. 2026 Jan 21;16:5908. doi: 10.1038/s41598-026-35990-9 (PMC12894976; doi:10.1038/s41598-026-35990-9)
Supplement: Supplementary file 2 — Supplementary Material 2 [file 41598_2026_35990_MOESM2_ESM.zip › AllTheTPPData/NvD40/Panobinostat_Vignette/Melting_Curves/meltCurve_Q0KCY2.pdf]

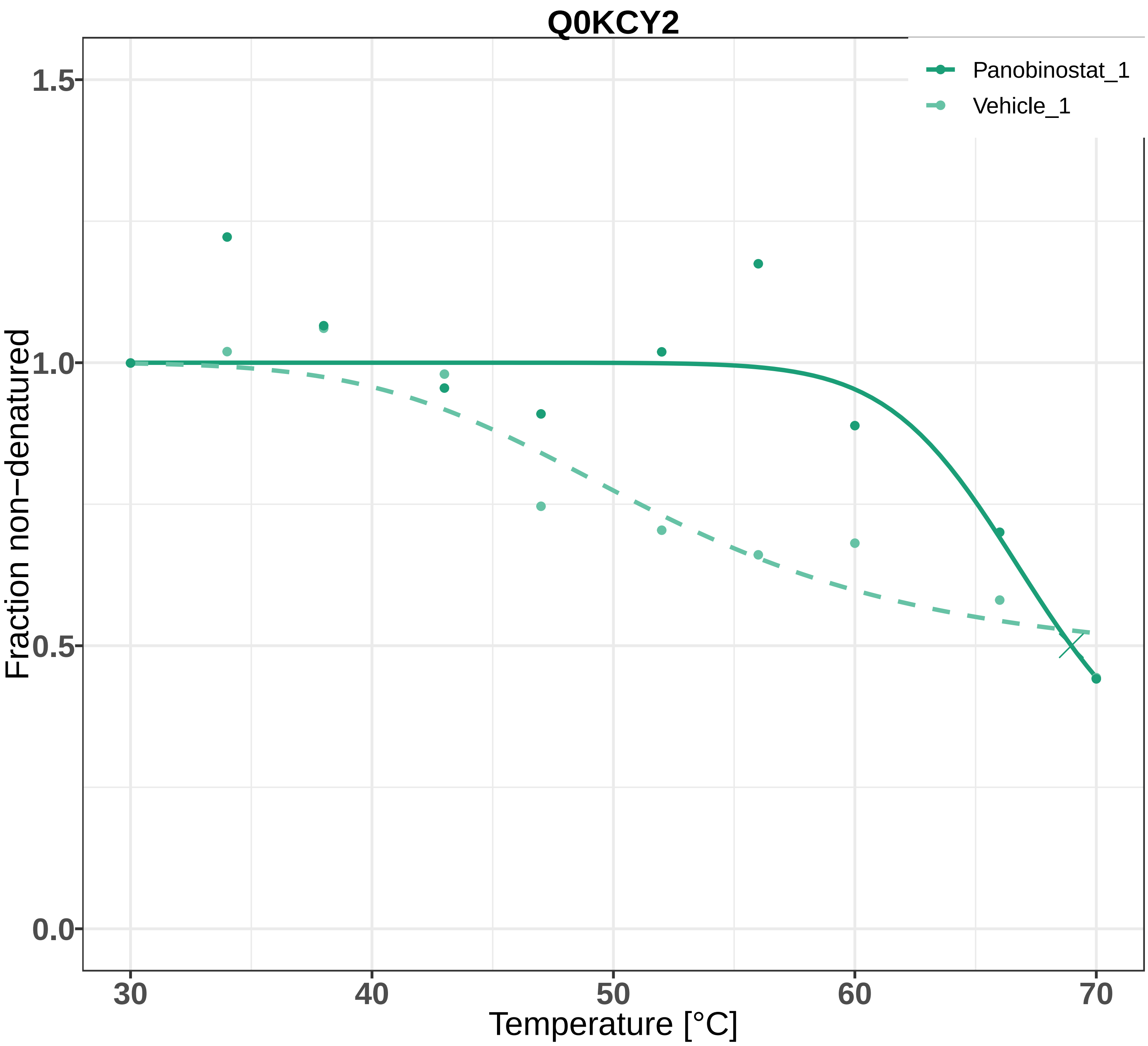


| | meltPoint | slope | plateau | R2 |
|---|---|---|---|---|
| Panobinostat_1 | 68.97 | −0.067 | 0.22 | 0.78 |
| Vehicle_1 | – | −0.022 | 0.46 | 0.91 |
